# Susceptibility patterns and the role of extracellular DNA in *Staphylococcus epidermidis* biofilm resistance to physico-chemical stress exposure

**DOI:** 10.1186/s12866-018-1183-y

**Published:** 2018-05-02

**Authors:** Charles Ochieng’ Olwal, Paul Oyieng’ Ang’ienda, David Miruka Onyango, Daniel Otieno Ochiel

**Affiliations:** Department of Zoology, School of Physical and Biological Sciences, Maseno University, P.O. Box, 333-40105, Maseno, Kenya

**Keywords:** *Staphylococcus epidermidis*, Bacterial biofilm, Susceptibility patterns, Extracellular DNA, Physico-chemical stresses

## Abstract

**Background:**

Over 65% of human infections are ascribed to bacterial biofilms that are often highly resistant to antibiotics and host immunity. *Staphylococcus epidermidis* is the predominant cause of recurrent nosocomial and biofilm-related infections. However, the susceptibility patterns of *S. epidermidis* biofilms to physico-chemical stress induced by commonly recommended disinfectants [(heat, sodium chloride (NaCl), sodium hypochlorite (NaOCl) and hydrogen peroxide (H_2_O_2_)] in domestic and human healthcare settings remains largely unknown. Further, the molecular mechanisms of bacterial biofilms resistance to the physico-chemical stresses remain unclear. Growing evidence demonstrates that extracellular DNA (eDNA) protects bacterial biofilms against antibiotics. However, the role of eDNA as a potential mechanism underlying *S. epidermidis* biofilms resistance to physico-chemical stress exposure is yet to be understood. Therefore, this study aimed to evaluate the susceptibility patterns of and eDNA release by *S. epidermidis* biofilm and planktonic cells to physico-chemical stress exposure.

**Results:**

*S. epidermidis* biofilms exposed to physico-chemical stress conditions commonly recommended for disinfection [heat (60 °C), 1.72 M NaCl, solution containing 150 μL of waterguard (0.178 M NaOCl) in 1 L of water or 1.77 M H_2_O_2_] for 30 and 60 min exhibited lower log reductions of CFU/mL than the corresponding planktonic cells (*p* < 0.0001). The eDNA released by sub-lethal heat (50 °C)-treated *S. epidermidis* biofilm and planktonic cells was not statistically different (*p* = 0.8501). However, 50 °C-treated *S. epidermidis* biofilm cells released significantly increased eDNA than the untreated controls (*p* = 0.0098). The eDNA released by 0.8 M NaCl-treated *S. epidermidis* biofilm and planktonic cells was not significantly different (*p* = 0.9697). Conversely, 5 mM NaOCl-treated *S. epidermidis* biofilms exhibited significantly increased eDNA release than the corresponding planktonic cells (*p* = 0.0015). Further, the 50 μM H_2_O_2_-treated *S. epidermidis* biofilms released significantly more eDNA than the corresponding planktonic cells (*p* = 0.021).

**Conclusions:**

*S. epidermidis* biofilms were less susceptible to physico-chemical stress induced by the four commonly recommended disinfectants than the analogous planktonic cells. Further, *S. epidermidis* biofilms enhanced eDNA release in response to the sub-lethal heat and oxidative stress exposure than the corresponding planktonic cells suggesting a role of eDNA in biofilms resistance to the physico-chemical stresses.

## Background

A bacterial population exists either as planktonic (free-floating cells) or as biofilm [1]. Bacterial biofilm refers to a sessile cluster of bacterial cells encased in a self-originating extracellular matrix (ECM) [2] composed of polysaccharides, proteins, water, lipids and nucleic acids [1]. Bacterial biofilms are ubiquitous and cause over 65% of human infections [3]. Moreover, bacterial biofilms are less susceptible to antibiotics and host immunity [4, 5]. *Staphylococcus epidermidis* is a Gram-positive coagulase negative bacteria most commonly linked with nosocomial and biofilm-related infections [6]. *S. epidermidis* adopts a biofilm lifestyle that enables resistance to antibiotics and host immunity, which potentially could lead to severe conditions such as bacteremia and sepsis, and if left untreated could result in death [7]. Up to 80% of infections of medical implant devices such as central venous catheters, cardiac pacemakers, tissue fillers, intrauterine devices and joint prostheses are caused by *S. epidermidis* biofilms [8].

Several studies have compared the susceptibilities of biofilm and planktonic forms of bacterial species to various physico-chemical stresses. For instance, studies have reported that biofilm forms of *Mycoplasma bovis* [9], *Vibrio cholerae* O1 [10], *Salmonella enterica* [11], *Burkholderia cenocepacia* [12], *Lactobacillus plantarum* subsp. *planturum* [13] *Pseudomonas aeruginosa* [14], *Mycobacterium avium*, *Mycobacterium intracellulare* [15] and *Klebsiella pneumoniae* [16] are more resistant to various conditions of heat, osmotic and oxidative stress exposure than the respective planktonic forms. In contrast to the bacterial species above, *S. epidermidis* biofilm is the most clinically relevant and a model of bacterial biofilm infections [17]. However, the susceptibility patterns of *S. epidermidis* biofilms to physico-chemical stresses commonly recommended for disinfection of food, drinking water, surfaces and medical equipment in domestic and human healthcare settings remains largely unknown.

The molecular mechanisms underlying bacterial biofilms resistance to stress agents remains largely unclear [18]. It is thought that mechanisms such as limited diffusion across the ECM barrier, the slow growth rate of biofilm cells, overproduction of antibiotics’ neutralizing enzymes, physiological heterogeneity of biofilms, the presence of persister cells and adaptive stress responses contribute to the high bacterial biofilm resistance [1, 3] against antimicrobials. However, the above mechanisms not only inconclusively explain the resistance of bacterial biofilms to antibiotics but also apply to a limited bacterial species [1, 6]. Growing evidence is beginning to link extracellular DNA (eDNA) of genomic origin released via active secretion or controlled cell lysis [19] with microbial biofilm resistance to various stress agents. For instance, eDNA has been shown to protect *S. epidermidis*, *Staphylococcus aureus* and *Actinobacillus pleuropneumoniae* biofilms against antibiotics such as vancomycin, β-lactams and penicillin G respectively [20–22]. Furthermore, a study reported that eDNA protects *Candida albicans* biofilm against 5 mM hydrogen peroxide (H_2_O_2_) stress [23]. The release of eDNA via lysis of a subpopulation of cells is a common phenomenon throughout the life cycle of biofilm-forming bacteria such as *S. epidermidis* [19]. However, the role that the released eDNA plays in *S. epidermidis* biofilms resistance to physico-chemical stress exposure is yet to be understood.

Therefore, the present study aimed to evaluate the susceptibility of and the eDNA release by *S. epidermidis* biofilm and planktonic cells to physico-chemical stress exposure.

## Methods

### Sample collection, bacterial isolate and growth conditions

The study was conducted between November 2015 and January 2017. *S. epidermidis* isolates were obtained by swabbing arm joints of seventy-one outpatients at Kisumu County Hospital, a referral health facility in western Kenya using a protocol described in [24]. A swab from each outpatient was plated on mannitol salt agar (MSA) (HiMedia Laboratories Pvt. Limited, Nashik, India) and then incubated for 24 h (hours) at 37 °C. One *S. epidermidis* isolate (largest colony) from the MSA plate was inoculated into 2 mL Tryptic Soy Broth (TSB) (Sigma Aldrich Chemie GmbH, Steinheim, Germany) and incubated at 37 °C with shaking at 120 revolutions per min (rpm) for 18 h to form *S. epidermidis* suspension. Biofilm-forming ability of each of the *S. epidermidis* suspension was assessed using the tube method biofilm assay as described in [25]. Only sixty-two *S. epidermidis* suspensions that were capable of forming biofilms were used in the present study.

A pair of *S. epidermidis* biofilm and planktonic cultures was generated from the biofilm-forming suspension as described in [26] with few modifications. Briefly, to generate *S. epidermidis* planktonic culture, 100 μL of the *S. epidermidis* suspension was inoculated into 10 mL fresh TSB (Sigma Aldrich Chemie GmbH, Steinheim, Germany) in conical polystyrene tube and then incubated at 37 °C with shaking at 120 rpm for 18 h. To generate *S. epidermidis* biofilm culture, 100 μL of the *S. epidermidis* suspension was transferred to a conical polystyrene tube containing 10 mL fresh TSB (Sigma Aldrich Chemie GmbH, Steinheim, Germany) supplemented with 1% glucose (Unilab Limited, Nairobi, Kenya) to induce biofilm formation and then incubated at 37 °C with shaking at 120 rpm for 24 h. In this study, *S. aureus* American Type Culture Collection (ATCC) 29,213 was used as a reference strain due to its good biofilm-forming ability within 24 h [27].

### Susceptibilities of *S. epidermidis* biofilm and planktonic cells to physico-chemical stress induced by the commonly recommended disinfectants

Based on the recommended guidelines for routine disinfection in domestic and human healthcare settings, the following physico-chemical stress conditions were used: heat (60 °C) [28], 1.72 M sodium chloride (NaCl) [29], 150 μL of waterguard in 1 L of water [30] and 1.77 M H_2_O_2_ [31]. Waterguard contains 0.178 M sodium hypochlorite (NaOCl) as the disinfectant [30].

### Procedure of exposure of *S. epidermidis* biofilm and planktonic cells to the physico-chemical stresses commonly recommended for disinfection

The effectiveness of the physico-chemical stress induced by the commonly recommended disinfectants against *S. epidermidis* biofilm and planktonic cells was determined as described in [16] with few modifications. Briefly, 1 mL of *S. epidermidis* biofilm or planktonic culture diluted to an OD_600_ of 1.0 was transferred to 9 mL of 1.72 M NaCl (Unilab Limited, Nairobi, Kenya), solution containing 150 μL waterguard in 1 L of water (Supersleek, Nairobi, Kenya) or 1.77 M H_2_O_2_ (RFCL Limited, New Delhi, India) vortexed for 2 min then incubated at 37 °C with shaking at 120 rpm for 60 min. For heat stress, 1 mL of *S. epidermidis* biofilm or planktonic culture diluted to an OD_600_ of 1.0 was added to 9 mL of sterile distilled water and then placed in a water bath model JSWB-11(T) (JS Research Inc., Gongju-city, Korea) at 60 °C. At 0, 30 and 60 min of exposure to each disinfectant, 1 mL sample was drawn for colony-forming units (CFUs) enumeration. To neutralize the waterguard and H_2_O_2_-treated cultures, 2 g/L sodium thiosulphate (Unilab Limited, Nairobi, Kenya) was placed in the first dilution tube. For NaCl stress-treated cultures, sterile distilled water was used instead of sodium thiosulphate. For heat stress, sterile water at 4 °C was placed in the first dilution tube to lower the temperature. Three replicate experiments were conducted.

### Enumeration and normalization of CFUs of *S. epidermidis* biofilm and planktonic cells exposed to the physico-chemical stresses commonly recommended for disinfection

The *S. epidermidis* biofilm and planktonic cells sampled at 0, 30 and 60 min were enumerated as described in [32] with few modifications. Briefly, 1 mL sample obtained at each time point (0, 30 and 60 min) was serially diluted 5-fold. Then, 100 μL of the 10^− 5^ dilution was plated in duplicate on Nutrient agar (HiMedia Laboratories Pvt. Limited, Mumbai, India) and then incubated for 20 h at 37 °C. The CFUs were counted using Colony Counter SC6 plus (Bibby Scientific Limited, Staffordshire, United Kingdom) and then converted into CFU/mL.

The CFU/mL values were normalized into log reduction of CFU/mL as described in [16]. Briefly, log reduction is defined as the negative log_10_ of the quotient of CFU after treatment and before treatment [−log_10_ (CFU_after treatment_ / CFU_before treatment_)]. The log reduction of CFU/mL for three replicate experiments were averaged and standard error of the mean (SEM) calculated. A log reduction value is directly proportional to the difference between the number of CFUs after and before treatment.

### Quantification of the effects of sub-lethal physico-chemical stress exposure on eDNA release by *S. epidermidis* biofilm and planktonic cells

It is recommended that bacterial biofilm samples exhibiting high resistance to disinfectants should be selected for further molecular analyses of the resistance mechanism(s) [33]. Therefore, a subset of *S. epidermidis* biofilm cultures (*n* = 12) that showed high resistance (smaller log reduction of CFU/mL units) to physico-chemical stress induced by the four commonly recommended disinfectants and the corresponding planktonic cultures were selected for eDNA experiments. Sub-lethal stress exposure induces bacterial eDNA release by partial repairable cell lysis or lysis-independent mechanisms and not via mass cell die off or explosive lysis [34] hence suitable for evaluating the effects of a stress agent on bacterial biofilm mechanisms such as eDNA release.

### Determination of the sub-lethal physico-chemical stress conditions

Sub-lethal concentrations of NaCl, NaOCl, and H_2_O_2_ were determined as described in [35] with some modifications. Briefly, 200 μL aliquot of *S. epidermidis* planktonic culture (pooled together from five different planktonic cultures) diluted to an OD_600_ of 1.0 was inoculated into 2 mL of increasing concentrations of NaCl (Unilab Limited, Nairobi, Kenya), NaOCl (Supersleek, Nairobi, Kenya) or H_2_O_2_ (RFCL Limited, New Delhi, India) for 60 min. At the 60th min, 1 mL of NaOCl and H_2_O_2_ stress-treated *S. epidermidis* cultures were neutralized with 200 μL of 2 g/L sodium thiosulphate (Unilab Limited, Nairobi, Kenya) and then serially diluted 8-fold. A 100 μL of the 10^− 8^ dilution was plated in duplicate on Tryptic Soy Agar (Sigma Aldrich Chemie GmbH, Steinheim, Germany) at 37 °C for 18 h. Then, CFUs were enumerated using Colony Counter SC6 plus (Bibby Scientific Limited, Staffordshire, United Kingdom). The following physico-chemical stress concentrations that induced stress without severe growth inhibition were used: 0.8 M NaCl, 5 mM NaOCl and 50 μM H_2_O_2_. For sub-lethal heat stress, 50 °C that has been shown to induce stress without degrading eDNA was used [36].

### Procedure of exposure of *S. epidermidis* biofilm and planktonic cells to the sub-lethal physico-chemical stress conditions

The *S. epidermidis* biofilm and planktonic cells were challenged with the four sub-lethal physico-chemical stresses as described in [16] with slight modifications. Briefly, 200 μL of *S. epidermidis* biofilm or planktonic culture diluted to an OD_600_ of 1.0 was inoculated into 400 μL of TSB (Sigma Aldrich Chemie GmbH, Steinheim, Germany) supplemented with 700 μL of 0.8 M NaCl (Unilab Limited, Nairobi, Kenya), 5 mM NaOCl (Supersleek, Nairobi, Kenya) or 50 μM H_2_O_2_ (RFCL Limited, New Delhi, India), vortexed for 2 min and then incubated at 37 °C with shaking at 120 rpm for 60 min. At the 60th min, NaOCl and H_2_O_2_ stress-treated *S. epidermidis* biofilm and planktonic cultures were neutralized by adding 200 μL of 2 g/L sodium thiosulphate (Unilab Limited, Nairobi, Kenya) to the tubes. For *S. epidermidis* biofilm or planktonic cultures exposed to 0.8 M NaCl, sterile distilled water was added instead of sodium thiosulphate. The untreated controls for NaCl, NaOCl and H_2_O_2_ comprised of 200 μL of *S. epidermidis* biofilm or planktonic culture inoculated into 400 μL of TSB (Sigma Aldrich Chemie GmbH, Steinheim, Germany) supplemented with 700 μL of sterile distilled water. For heat stress exposure, 200 μL of *S. epidermidis* biofilm or planktonic culture was inoculated into 400 μL of TSB (Sigma Aldrich Chemie GmbH) supplemented with 700 μL of sterile distilled water and then transferred to a water bath model JSWB-11(T) (JS Research Inc., Gongju-city, Korea) at 50 °C for 60 min. At the 60th min, 200 μL of sterile distilled water at 4 °C was added to lower the temperature. The untreated control was 200 μL of *S. epidermidis* biofilm or planktonic culture inoculated into 400 μL of TSB (Sigma Aldrich Chemie GmbH) supplemented with 700 μL of sterile distilled water then transferred to water bath model JSWB-11(T) (JS Research Inc., Gongju-city, Korea) at 25 °C for 60 min.

### Isolation of eDNA

To minimize variations associated with DNA precipitation, eDNA was obtained directly from the supernatant [37]. The exopolymeric substances were separated from bacterial cells by high-speed centrifugation which does not cause cell lysis [38]. Free and bound eDNA were then obtained from the exopolymeric substances using Tris-Ethylenediaminetetraacetic acid (EDTA) (TE) buffer [39, 40].

The eDNA released by the sub-lethal stress-treated *S. epidermidis* biofilm and planktonic cultures and their respective untreated controls was obtained as described in [20] with few modifications. Briefly, sub-lethal physico-chemical stress-treated culture or untreated control was centrifuged at 20,000 rpm at 4 °C for 20 min. Then, 1 mL of the supernatant was pipetted into 1 mL of TE buffer (10 mM Tris, 1 mM EDTA, pH 8.0) and then centrifuged at 13,000 rpm for 3 min. Finally, 30 μL of the supernatant was suspended in 100 μL of TE buffer and then stored at − 20 °C until further use.

### Quantification of eDNA

The eDNA in the supernatant was quantified using Qubit™ dsDNA high sensitivity (HS) assay kit (Invitrogen, Paisley, United Kingdom) and Qubit® 2.0 Fluorometer (Life Technologies, Carlsbad, USA) following the manufacturers’ instructions. Briefly, Qubit working solution was prepared by diluting 1 μL of Qubit™ dsDNA HS reagent (Molecular Probes Inc., Willow Creek Road Eugene, Oregon) with 199 μL of Qubit™ dsDNA HS buffer (Invitrogen, Paisley, United Kingdom) in a plastic tube. Then, 2 μL of the supernatant was added to 198 μL of the working solution in a plastic tube, vortexed for 3 s and then incubated at room temperature for 2 min. The tube was loaded into a Qubit® 2.0 Fluorometer (Life Technologies, Carlsbad, USA) to quantify eDNA in ng/μL. The experiments were performed in duplicate. Percentage change in eDNA yield expressed as [{eDNA_stress treated cells_ – eDNA_untreated controls_) / eDNA_untreated controls_} × 100%] was computed for pairs of *S. epidermidis* biofilm and planktonic cultures.

### Data analyses

Data obtained were stored in Microsoft Office Excel and analyzed using Prism 5 for windows version 5.03 (GraphPad software, Inc., California, USA). Data normality was verified using Shapiro–Wilk test. Normally and non-normally distributed data were presented as mean (± SEM) and median (25th and 75th percentiles) respectively. Differences in log reduction of CFU/mL between *S. epidermidis* biofilm and planktonic cells subjected to each of the four commonly recommended physico-chemical disinfectants were compared by paired *t*-test. Comparisons of log reductions of CFU/mL of *S. epidermidis* biofilm or planktonic cells among the four commonly recommended physico-chemical disinfectants were performed using repeated measures analysis of variance (ANOVA) with Tukey’s post hoc. Differences in eDNA release between *S. epidermidis* biofilm and planktonic cells treated with each of the four sub-lethal physico-chemical stresses were conducted by Wilcoxon matched-pairs signed rank test. Similarly, differences in eDNA yield between sub-lethal physico-chemical stress-treated *S. epidermidis* biofilm or planktonic cells and their respective untreated controls were analyzed by Wilcoxon matched-pairs signed rank test. Statistical significance was considered at *p* < 0.05.

## Results

### Susceptibility patterns of *S. epidermidis* biofilm and planktonic cells to physico-chemical stress induced by the commonly recommended disinfectants

One of the specific aims of the present study was to determine the susceptibility patterns of *S. epidermidis* biofilm and planktonic cells to physico-chemical stress induced by the four commonly recommended disinfectants (heat, NaCl, NaOCl and H_2_O_2_) in domestic and human healthcare settings.

### *S. epidermidis* planktonic cells are more susceptible to heat stress than the biofilm cells

**Fig. 1:**
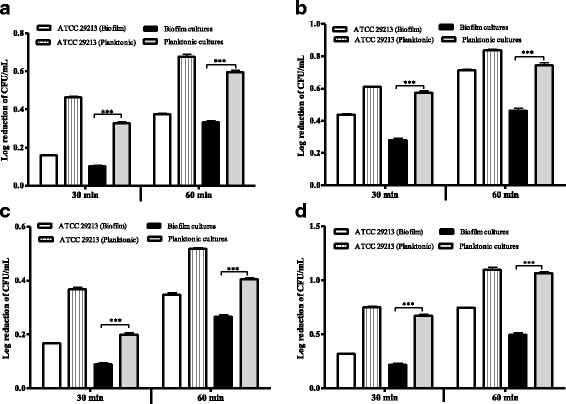
Susceptibility patterns of *S. epidermidis* biofilm and planktonic cells to physico-chemical stress exposure. The log reduction of CFU/mL of pairs of *S. epidermidis* biofilm and planktonic cultures challenged with 60 °C (**a**), 1.72 M NaCl (**b**), a solution containing 150 μL of waterguard in 1 L of water (**c**) and 1.77 M H_2_O_2_ (**d**) for 30 and 60 min. Value for ATCC 29213 represents the mean for three independent experiments. Error bars depict SEM. Statistical significance between *S. epidermidis* biofilm and planktonic cultures were determined using paired *t*-test (***, *p* < 0.0001)

The *S. epidermidis* biofilms exposed to heat (60 °C) stress for 30 min exhibited a significantly lower log reduction of CFU/mL than the corresponding planktonic cells (*p* < 0.0001) (Fig. 1a). Similarly, the log reduction of CFU/mL of *S. epidermidis* biofilms subjected to the same stress for 60 min was statistically lower than the planktonic cells (*p* < 0.0001) (Fig. 1a). These results indicated that 60 °C was less effective against *S. epidermidis* biofilms compared to the corresponding planktonic cells. Further analyses showed that *S. epidermidis* biofilm or planktonic cells subjected to 60 °C stress for 30 min had a significantly lower log reduction of CFU/mL compared to their respective cells exposed to 60 °C stress for 60 min (*p* < 0.0001) (Table 1). This indicated that the *S. epidermidis* biofilm or planktonic cells killing by the heat stress were directly proportional to the exposure duration.

**Table 1:** Effectiveness of physico-chemical stress exposure durations on *S. epidermidis* biofilm and planktonic cells

Type of culture	Mean ± SEM of log reduction of CFU/mL of cells exposed to commonly recommended stresses			
	Heat	NaCl	NaOCl	H_2_O_2_
Biofilm				
30 min	0.110 ± 0.006	0.281 ± 0.011	0.089 ± 0.005	0.219 ± 0.011
60 min	0.332 ± 0.009	0.464 ± 0.014	0.266 ± 0.007	0.494 ± 0.018
	*p* < 0.05	*p* < 0.05	*p* < 0.05	*p* < 0.05
Planktonic				
30 min	0.342 ± 0.007	0.574 ± 0.013	0.199 ± 0.007	0.673 ± 0.013
60 min	0.596 ± 0.010	0.744 ± 0.015	0.404 ± 0.007	1.067 ± 0.013
	*p* < 0.05	*p* < 0.05	*p* < 0.05	*p* < 0.05

*S. epidermidis* biofilm and planktonic cultures were exposed to 60 °C, 1.72 M NaCl, a solution containing 150 μL of waterguard in 1 L of water and 1.77 M H_2_O_2_ for 30 and 60 min. Statistical significance between 30 and 60 min physico-chemical stress exposure durations on *S. epidermidis* biofilm or planktonic cultures was determined by paired *t*-test

### *S. epidermidis* biofilms are less susceptible to NaCl stress than the planktonic cells

When challenged with 1.72 M NaCl for 30 min, *S. epidermidis* biofilms exhibited a significantly lower log reduction of CFU/mL than the analogous planktonic cells (*p* < 0.0001) (Fig. 1b). Treatment of *S. epidermidis* biofilm and planktonic cells with 1.72 M NaCl for 60 min yielded a similar pattern (*p* < 0.0001) (Fig. 1b). These results showed that 1.72 M NaCl was less effective against *S. epidermidis* biofilms than the corresponding planktonic cells. Further analyses revealed that *S. epidermidis* biofilm or planktonic cells subjected to 1.72 M NaCl stress for 30 min had a significantly lower log reduction of CFU/mL than their respective cells exposed to 1.72 M NaCl stress for 60 min (*p* < 0.0001) (Table 1). These implied that the effectiveness of 1.72 M NaCl on *S. epidermidis* biofilm or planktonic cells is dependent on the exposure duration.

### NaOCl stress is more effective against *S. epidermidis* planktonic than biofilm cells

*S. epidermidis* biofilms exhibited significantly lower log reduction of CFU/mL when exposed to a solution containing 150 μL of waterguard in 1 L of water for 30 min than the analogous planktonic cells (*p* < 0.0001) (Fig. 1c). A similar pattern was observed upon exposure of *S. epidermidis* biofilm and planktonic cells to a solution containing 150 μL waterguard in 1 L of water for 60 min (*p* < 0.0001) (Fig. 1c). These results indicated that *S. epidermidis* biofilms were more protected against a solution containing 150 μL of waterguard in 1 L of water than the corresponding planktonic cells. Further analyses showed that *S. epidermidis* biofilm or planktonic cells subjected to a solution containing 150 μL of waterguard in 1 L of water for 30 min had a significantly lower log reduction of CFU/mL than their respective cells exposed to the same stress for 60 min (*p* < 0.0001) (Table 1). These results implied that the effectiveness of NaOCl stress against *S. epidermidis* biofilm and planktonic cells was proportional to the exposure duration.

### H_2_O_2_ stress is less effective against *S. epidermidis* biofilms than planktonic cells

*S. epidermidis* biofilms treated with 1.77 M H_2_O_2_ for 30 min had a statistically lower log reduction of CFU/mL than the analogous planktonic cells (*p* < 0.0001) (Fig. 1d). Similarly, *S. epidermidis* biofilms challenged with 1.77 M H_2_O_2_ for 60 min showed a significantly lower log reduction than the corresponding planktonic cells (*p* < 0.0001) (Fig. 1d). These results indicated that 1.77 M H_2_O_2_ stress is more effective against *S. epidermidis* planktonic cells than the corresponding biofilm cells. Further, *S. epidermidis* biofilm or planktonic cells challenged with 1.77 M H_2_O_2_ for 30 min showed a significantly lower log reduction of CFU/mL than their respective cells subjected to the same stress for 60 min (*p* < 0.0001) (Table 1). This implied that the efficacy of 1.77 M H_2_O_2_ against *S. epidermidis* biofilm and planktonic cells was directly proportional to the exposure duration.

### Comparison of the effectiveness of physico-chemical stress induced by the commonly recommended disinfectants against *S. epidermidis* biofilm and planktonic cells

**Fig. 2:**
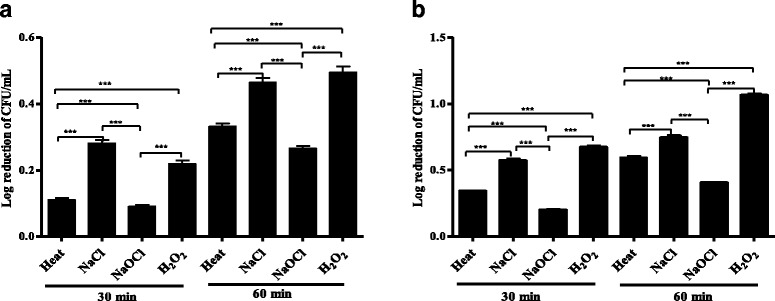
Comparison of the effectiveness of physico-chemical stresses against *S. epidermidis* biofilm and planktonic cells. The log reduction of CFU/mL of pairs of *S. epidermidis* biofilm (**a**) and planktonic (**b**) cultures subjected to 60 °C, 1.72 M NaCl, a solution containing 150 μL of waterguard in 1 L of water and 1.77 M H_2_O_2_ for 30 and 60 min. Error bars represent SEM. Statistical significance was determined using repeated measures ANOVA with Tukey’s post hoc (***, *p* < 0.0001)

The present study also compared the effectiveness of physico-chemical stress induced by the four commonly recommended disinfectants against *S. epidermidis* biofilm or planktonic cells. Repeated measures ANOVA showed that the log reductions of CFU/mL of *S. epidermidis* biofilm cells differed significantly among the four commonly recommended disinfectants upon exposure for 30 or 60 min (*p* < 0.0001). Tukey’s post hoc revealed that when *S. epidermidis* biofilm cells are exposed to the four commonly recommended disinfectants for 30 min, the log reduction of CFU/mL was significantly highest for 1.72 M NaCl followed by 1.77 M H_2_O_2_, 60 °C and a solution containing 150 μL of waterguard in 1 L of water in that order (Fig. 2a). Conversely, when *S. epidermidis* biofilms were subjected to the four commonly recommended disinfectants for 60 min, Tukey’s post hoc showed that the log reduction of CFU/mL was significantly highest for 1.77 M H_2_O_2_, followed by 1.72 M NaCl, 60 °C and a solution containing 150 μL of waterguard in 1 L of water in that order (Fig. 2a). These results indicated that susceptibilities of *S. epidermidis* biofilm cells to physico-chemical stress induced by the four commonly recommended disinfectants for 30 min was not dependent on the diffusion rate (molecular weight) (NaCl > H_2_O_2_ > heat > NaOCl). However, susceptibility of *S. epidermidis* biofilm cells to physico-chemical stress induced by the four commonly recommended disinfectants for 60 min was dependent on the diffusion rate of the disinfectants (H_2_O_2_ > NaCl > heat > NaOCl).

Furthermore, repeated measures ANOVA revealed that the log reductions of CFU/mL of *S. epidermidis* planktonic cells differed significantly among the four commonly recommended disinfectants upon exposure for 30 or 60 min (*p* < 0.0001). Tukey’s post hoc showed that the log reduction of CFU/mL of *S. epidermidis* planktonic cells were significantly highest for 1.77 M H_2_O_2_, followed by 1.72 M NaCl, 60 °C and a solution containing 150 μL of waterguard in 1 L of water in that order at both 30 and 60 min of exposure (H_2_O_2_ > NaCl > heat > NaOCl) (Fig. 2b). These results implied that the susceptibilities of *S. epidermidis* planktonic cells to the four commonly recommended disinfectants for 30 or 60 min were dependent on the diffusion rate of the disinfectants.

### Effects of sub-lethal physico-chemical stress exposure on eDNA release by *S. epidermidis* biofilm and planktonic cells

To effectively control, manage and eradicate bacterial biofilms in domestic and healthcare settings it is necessary to understand the mechanisms that mediate their survival against commonly reccommded physico-chemical disinfectants. Consequently, a second specific aim of the present study was to evaluate the eDNA release as a potential mechanism that underlies *S. epidermidis* biofilms resistance to physico-chemical stress exposure.

### Effect of heat stress on eDNA release by *S. epidermidis* biofilm and planktonic cells

**Fig. 3:**
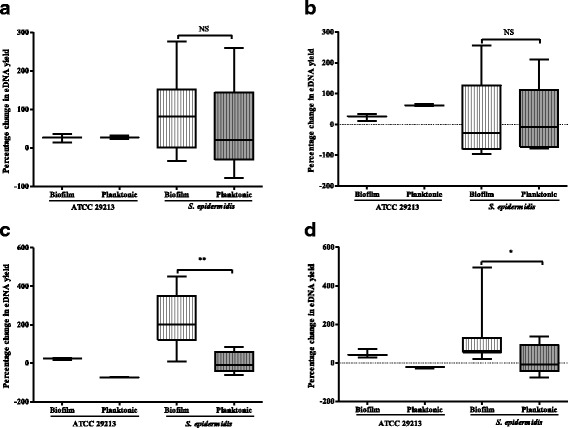
Impacts of physico-chemical stress exposure on eDNA release by *S. epidermidis* biofilm and planktonic cells. The percentage change in eDNA yield by pairs of *S. epidermidis* biofilm and planktonic cultures exposed to sub-lethal heat (50 °C) (**a**), 0.8 M NaCl (**b**), 5 mM NaOCl (**c**) and 50 μM H_2_O_2_ (**d**) for 60 min. The horizontal line across the box is the median percentage change in eDNA yield, the lower and upper ends of the box are the 25th and 75th percentiles. The whiskers represent the minimum and maximum percentage changes in eDNA yield. Values for ATCC 29213 are for three independent experiments. Statistical significance between *S. epidermidis* biofilm and planktonic cultures were determined by Wilcoxon matched-pairs signed rank test (^NS^, *p* > 0.05; *, *p* < 0.05; **, *p* < 0.01)

The percentage change in eDNA yield by 50 °C-treated *S. epidermidis* biofilms was slightly more than the corresponding planktonic cells albeit not statistically different (*p* = 0.4697) (Fig. 3a). Further analysis showed that 50 °C-treated *S. epidermidis* biofilm cells released significantly increased eDNA than the respective untreated controls (*p* = 0.0098) (Table 2). On the contrary, 50 °C-treated planktonic cells yielded more eDNA than the untreated controls but the difference was not statistically significant (*p* = 0.7910) (Table 2).

**Table 2:** Impacts of sub-lethal physico-chemical stresses on eDNA release by *S. epidermidis* biofilm and planktonic cells

Type of culture	Mean ± SEM of eDNA yield in ng/μL by cells exposed to sub-lethal stresses			
	Heat	NaCl	NaOCl	H_2_O_2_
Biofilm				
Untreated controls	0.110 ± 0.019	0.482 ± 0.159	0.089 ± 0.021	0.231 ± 0.056
Treated cells	0.221 ± 0.058	0.285 ± 0.095	0.292 ± 0.072	0.451 ± 0.095
	*p = 0.0098*	*p = 0.3271*	*p = 0.0005*	*p = 0.0005*
Planktonic				
Untreated controls	0.405 ± 0.101	0.242 ± 0.067	0.526 ± 0.128	0.137 ± 0.031
Treated cells	0.394 ± 0.071	0.219 ± 0.089	0.536 ± 0.123	0.112 ± 0.015
	*p* = 0.7910	*p* = 0.6772	*p* = 0.9097	*p* = 0.7910

*S. epidermidis* biofilm and planktonic cultures were subjected to heat (25 and 50 °C), NaCl (0 and 0.8 M), NaOCl (0 and 5 mM) and H_2_O_2_ (0 and 50 μM) for 60 min. Italics indicate statistical significance (*p* < 0.05) between stress treated cultures and the untreated controls as determined by Wilcoxon matched-pairs signed rank test

### Impact of NaCl stress on eDNA release by *S. epidermidis* biofilm and planktonic cells

*S. epidermidis* planktonic cells exposed to 0.8 M NaCl stimulated higher percentage change in eDNA yield than the analogous biofilm cells although the difference was not statistically significant (*p* = 0.8501) (Fig. 3b). Further analysis showed that 0.8 M NaCl-treated *S. epidermidis* biofilm cells released less eDNA than the untreated controls although not statistically different (*p* = 0.3804) (Table 2). However, 0.8 M NaCl-treated *S. epidermidis* planktonic cells yielded more eDNA than the untreated controls but the difference was not statistically significant (*p* = 0.6772) (Table 2). These results showed that osmotic stress does not significantly affect eDNA release by *S. epidermidis* biofilm and planktonic cells.

### Effects of oxidative stresses on eDNA release by *S. epidermidis* biofilm and planktonic cells

Significantly increased percentage change in eDNA was released by *S. epidermidis* biofilms exposed to 5 mM NaOCl compared to the corresponding planktonic cells (*p* = 0.0015) (Fig. 3c). Further analysis showed that 5 mM NaOCl-treated *S. epidermidis* biofilm cells released significantly more eDNA than the untreated controls (*p* = 0.0005) (Table 2). On the contrary, 5 mM NaOCl-treated *S. epidermidis* planktonic cells produced slightly more eDNA than the untreated controls but the difference was not significant (*p* = 0.9097) (Table 2).

When challenged with 50 μM H_2_O_2_ stress for 60 min, the *S. epidermidis* biofilm cells exhibited significantly increased percentage change in eDNA than the analogous planktonic cells (*p* = 0.0210) (Fig. 3d). Further analyses revealed that 50 μM H_2_O_2_-treated *S. epidermidis* biofilm cells produced significantly more eDNA than the untreated controls (*p* = 0.0005) (Table 2). In contrast, the eDNA yield between 50 μM H_2_O_2_-treated *S. epidermidis* planktonic cells and the respective untreated controls was not significantly different (*p* = 0.7910) (Table 2). Taken together, these results indicated that *S. epidermidis* biofilm cells enhance eDNA release in response to 5 mM NaOCl and 50 μM H_2_O_2_ stress exposure.

## Discussion

### Susceptibility patterns of *S. epidermidis* biofilm and planktonic cells to physico-chemical stress induced by the commonly recommended disinfectants

Bacterial biofilms are most frequently encountered in domestic and healthcare environments [41, 42]. Thus, prevention of acquisition, spread and establishment of biofilm-forming bacteria such as *S. epidermidis* using effective disinfection guidelines [12] in these environments is necessary. Therefore, the present study evaluated the effectiveness of four commonly recommended physico-chemical disinfectants against *S. epidermidis* biofilm and planktonic cells.

The present study showed that the commonly recommended heat stress was less effective against *S. epidermidis* biofilms compared to the corresponding planktonic forms. The present findings are in agreement with previous reports on different bacterial species such as *M. bovis* [9], *S*. *enterica* [11] and a fungus, *Cryptococcus neoformans* [43]. However, the previous studies reported on different bacterial species such as *M. bovis* which are cell wall-less, *S. enterica* that overproduce protective cellulosic polymer and a fungal biofilm hence may not inform bacterial biofilm response to heat stress. The observed increased heat stress resistance of *S. epidermidis* biofilms compared to the corresponding planktonic cells could probably be explained in two ways. First, since bacterial biofilms overexpress heat stress-related genes [44] resulting in heat shock proteins that consume excess energy in form of adenosine triphosphate [45], it is likely that the *S. epidermidis* biofilm cells overexpressed heat stress-related genes to protect them against the deleterious effects of heat stress. Second, an increase in temperature has been shown to switch the staphylococcal biofilm cells fatty acid profile such that the anteiso-C19 fatty acids known to have high melting point rises, leading to decreased membrane fluidity resulting in increased resistance to heat stress [32].

Furthermore, the present study showed that biofilm forms of *S. epidermidis* are more resistant to the commonly recommended NaCl stress than the analogous planktonic cells which is consistent with a previous report on *V*. *cholerae* O1 by Wai et al. [10]. However, the previous study used *V. cholerae* a bacterium highly adapted to high salinity [46] and utilized high salt concentration (2.5 M NaCl) that may not inform routine bacterial biofilm disinfection. The observed increased resistance of *S. epidermidis* biofilm cells to osmotic stress could possibly be due to the osmotic stress-enhanced production of exopolysaccharides and proteins that formed a water-retaining layer around *S. epidermidis* biofilm cells thus protecting the cells against dehydration [23, 47]. An alternative explanation could be linked to the previous observation that osmotic stress enhances quorum sensing in bacterial biofilms [48] which correspondingly increases resistance against osmotic stress [49] by the *S. epidermidis* biofilm cells.

The present study also showed that the commonly recommended NaOCl stress is less effective against *S. epidermidis* biofilms compared to the analogous planktonic cells. The present finding is in consonance with several previous reports on different bacterial species that were either overproducing protective pellicles [11], were subjected to high [13] or low NaOCl concentrations [12], were overgrown for six days [16] or had protective mycolic acid rich membranes [15]. Thus, the previous reports may not inform general bacterial biofilm response to NaOCl stress. The observed increased *S. epidermidis* biofilm resistance could partly be due to the reaction of NaOCl with the ECM components such as proteins and polysaccharides and/or the slow diffusion across ECM barrier thus reducing the effect of NaOCl on most *S. epidermidis* biofilm cells [16].

The present study also demonstrated that *S. epidermidis* biofilms are more protected against the commonly recommended H_2_O_2_ stress than the analogous planktonic cells. The present finding concurs with previous reports on *S. epidermidis* [50], *V. cholerae* [10] and *B. cenocepacia* [12] exposed to low concentrations of H_2_O_2_ that are not routinely used for disinfection. In addition, Glynn et al. [50] reported on the effect of H_2_O_2_ on *S. epidermidis* biofilm formation using a semiquantitative approach which may not present a clear picture of *S. epidermidis* biofilms response to H_2_O_2_ stress. The observed increased resistance of *S. epidermidis* biofilms could possibly be attributed to neutralization of H_2_O_2_ by the ECM components such as polysaccharides and proteins and/or overexpression of catalase-producing genes resulting in overproduction of catalase enzymes that decompose the H_2_O_2_ [12] thereby reducing its effects on most of the inner *S. epidermidis* biofilm cells.

Taken together, the results presented in the present study showed that the susceptibility of *S. epidermidis* biofilm and planktonic cells was proportional to the duration of exposure to each of the four commonly recommended disinfectants. Generally, a disinfectant producing a log reduction unit above three (99.9% bacterial reduction) is considered effective against a bacterial biofilm [33]. However, in the present study, *S. epidermidis* biofilm cells exhibited log reduction units below three (< 3) when subjected to the four commonly recommended disinfectants. This implied that the four physico-chemical stresses commonly recommended for disinfection in domestic and human healthcare settings were ineffective against the *S. epidermidis* biofilm cells hence creating a healthcare concern.

### Reaction-diffusion inhibition mechanism inadequately explains the increased *S. epidermidis* biofilms resistance to physico-chemical stresses commonly recommended for disinfection

Bacterial biofilm resistance to osmotic and oxidative stress exposure is mostly attributed to the stress agent’s reaction with and/or slow diffusion across the ECM (reaction-diffusion inhibition mechanism) [16, 51]. The movement of the stress agents probably occur via water-filled channels on the bacterial biofilm’s ECM [2]. The observation that the susceptibility of *S. epidermidis* biofilm cells to the commonly recommended disinfectants for 60 min depended on the diffusion rate (molecular weight) (H_2_O_2_ > NaCl > heat > NaOCl) appear to support the reaction-diffusion inhibition mechanism. It has been shown that NaOCl (with slowest diffusion rate) diffuses across the ECM in about 50 min [16]. Accordingly, all the four disinfectants should have crossed the ECM within the 60 min of exposure and killed an equal number of *S. epidermidis* biofilm and planktonic cells. Taking into account the observation that more *S. epidermidis* planktonic cells were killed compared to the corresponding biofilm cells at 60 min of exposure and that susceptibilities of the biofilm cells to the commonly recommended disinfectants for 30 min did not correspond to the diffusion rate, it is reasonable to surmise that the reaction-diffusion inhibition mechanism inadequately explains the increased resistance of *S. epidermidis* biofilm cells to the physico-chemical stress induced by the commonly recommended disinfectants. This lends credence to the existence of complementary mechanism(s) of resistance against physico-chemical stress exposure in *S. epidermidis* biofilms such as eDNA release [19] and upregulation of biofilm-specific stress resistance genes [3].

### Role of eDNA in *S. epidermidis* biofilms resistance to physico-chemical stress exposure

The reaction-diffusion inhibition mechanism did not fully account for the increased *S. epidermidis* biofilms resistance to physico-chemical stress induced by the commonly recommended disinfectants relative to the planktonic forms. In the recent years, some studies have linked eDNA release with survival of bacterial [20–22] and fungal biofilms [23] against antibiotics and H_2_O_2_ respectively. Thus, the present study evaluated eDNA release as a potential complementary mechanism underlying *S. epidermidis* biofilms resistance to physico-chemical exposure.

The present study did not reveal any significant difference in eDNA release between the *S. epidermidis* biofilm and planktonic forms exposed to sub-lethal heat stress. However, the present study showed that the sub-lethal heat stress-treated *S. epidermidis* biofilms significantly enhanced eDNA release than the untreated controls. For *S. epidermidis* planktonic cells, no difference was observed in eDNA release between the treated cells and untreated controls. This set of results can be interpreted in two ways. On the one hand, the finding that eDNA released by *S. epidermidis* biofilm and planktonic cells were not statistically different could mean that eDNA release was slightly stimulated by a rise in temperature via active secretion or controlled cell lysis [19] in both the biofilm and planktonic cells and not necessarily as a resistance mechanism against the heat stress exposure. On the other hand, the observation that unlike the planktonic forms, the biofilm forms of *S. epidermidis* subjected to heat stress released significantly increased eDNA than the untreated controls strongly suggested a central role of eDNA in *S. epidermidis* biofilm cells resistance against the heat stress.

Further, there was no difference between eDNA released by *S. epidermidis* biofilm and planktonic cells subjected to sub-lethal NaCl stress. Unexpectedly, the NaCl stress treated *S. epidermidis* biofilms released less eDNA than the untreated controls. A possible explanation for this unexpected observation could be inferred from previous studies which have shown that NaCl stress stimulates increased exopolysaccharide production in the ECM [23, 47] which might have resulted in strong bond formation between the eDNA and polyssacharides [52] rendering it largely inaccessible for quantification. Considered together, the present findings show that NaCl stress does not affect eDNA release by both *S. epidermidis* biofilm and planktonic cells. The present finding concurs with a previous report on *C. albicans* biofilm subjected to 2 M NaCl [23]. However, *C. albicans* is a fungus hence may not inform bacterial biofilms response to osmotic stress. Further, the present findings are consistent with a previous report which showed that high salt concentration affects exopolysaccharide release by *Halomonas variabilis* and *Planococcus rifietoensis* [47]. However, the previous report only evaluated one component of the ECM that is the exopolysaccharide. The observation that neither *S. epidermidis* biofilm and planktonic cells nor the respective treated and untreated controls showed a significant difference in eDNA release suggested that eDNA does not play a role in osmotic stress resistance. In support of this interpretation, a study showed that osmotic stress does not produce eDNA richer ECM instead, exopolysaccharide and protein yield is enhanced to form a water-retaining layer around the biofilm cells [23] thus protecting the cells against dehydration. Moreover, it has been shown that *autolysin* (*atl*) gene, which is often associated with eDNA release is not affected by osmotic stresses [53] further implying that osmotic stress does not induce eDNA release.

Further, the present study showed that *S. epidermidis* biofilms enhance eDNA release in response to sub-lethal oxidative (NaOCl and H_2_O_2_) stress exposure. This is consistent with previous reports, which showed that H_2_O_2_ stress exposure induced eDNA release by *Streptococcus gordonii* [34, 37] and *C. albicans* biofilm [23]. However, the previous reports only focused on planktonic forms of *S. gordonii* and a fungus, *C. albicans* hence may not inform eDNA release by bacterial biofilms in response to H_2_O_2_.

Considered together, oxidative stresses damage genomic DNA triggering eDNA release by a subpopulation of bacterial cells [37]. Why oxidative stress-treated *S. epidermidis* biofilms released more eDNA than the corresponding planktonic cells is still not clear. One possible explanation could be related to the extracellular DNases released alongside eDNA in the following ways. First, unlike the planktonic cells, bacterial biofilm cells form small-protected pockets [21] that could be protecting most eDNA from DNases degradation. Second, bacterial biofilms eDNA is mostly bound to ECM [52] hence may not be easily accessible to the DNases. Third, bacterial biofilms produce relatively fewer DNases than the planktonic cells [54] thus minimizing the eDNA degradation. Fourth, bacterial biofilms induce release of proteolytic exoenzymes that inactivate the DNases locally [55]. Taken together, the explanations above suggest that eDNA and DNases release by bacterial biofilm cells are highly regulated processes. This implies that bacterial planktonic cells majorly release eDNA to be degraded for nutrients whereas bacterial biofilm cells induce eDNA release both as a nutrient source and for protection against the lethal effects of oxidative stresses.

An alternative explanation for the relatively increased eDNA release by *S. epidermidis* biofilm cells could be related to the high production of catalase enzyme [56] and ECM [12, 57] which neutralizes and reacts with the H_2_O_2_ respectively. It has also been shown that NaOCl reacts with the organic components of the ECM thereby reducing its concentration [16]. Thus, the relatively increased eDNA release by *S. epidermidis* biofilm cells despite the potential exposure to lower concentrations of NaOCl and H_2_O_2_ strongly suggested an integral role of eDNA in the biofilm cells resistance to the oxidative stresses.

### Limitations

A potential limitation of the present study is that although DNase appears to provide a more plausible explanation for the increased eDNA release by *S. epidermidis* biofilms challenged with the sub-lethal oxidative stresses, the presence of DNase was not quantitatively measured. Further studies with DNase (+) controls and treatment groups may be necessary to confirm the direct link between eDNA and bacterial biofilm resistance to physico-chemical stresses.

## Conclusions

In summary, *S. epidermidis* biofilms were less susceptible to physico-chemical stress induced by the four commonly recommended disinfectants than the corresponding planktonic forms. Thus, there is need to review the current disinfection guidelines to improve *S. epidermidis* biofilm disinfection efficiency. Further, *S. epidermidis* biofilms enhanced eDNA release in response to sub-lethal heat and oxidative stress exposure than the corresponding planktonic cells, suggesting a role of eDNA in the bacterial biofilms resistance to physico-chemical stress exposure. Therefore, eDNA may be a potential target for novel anti-bacterial biofilm control and eradication strategies.

## Abbreviations

ATCC: American type culture collection
CFUs: Colony-forming units
dsDNA: Double-stranded DNA
ECM: Extracellular matrix
eDNA: Extracellular DNA
H_2_O_2_: Hydrogen peroxide
HS: High sensitivity
MSA: Mannitol salt agar
NaCl: Sodium chloride
NaOCl: Sodium hypochlorite
OD: Optical density
rpm: Revolutions per minute
SEM: Standard error of the mean
TE: Tris-Ethylenediaminetetraacetic acid
TSB: Tryptic soy broth
